# Rhythm on Your Lips

**DOI:** 10.3389/fpsyg.2016.01708

**Published:** 2016-11-08

**Authors:** Marcela Peña, Alan Langus, César Gutiérrez, Daniela Huepe-Artigas, Marina Nespor

**Affiliations:** ^1^Laboratorio de Neurociencias Cognitivas, Escuela de Psicología, Pontificia Universidad Católica de ChileSantiago, Chile; ^2^Language, Cognition and Development Lab, International School for Advanced StudiesTrieste, Italy; ^3^Center for Social and Cognitive Neuroscience, School of Psychology, Universidad Adolfo IbañezSantiago, Chile

**Keywords:** language, speech perception, visual perception, lip reading, iambic-trochaic law

## Abstract

The Iambic-Trochaic Law (ITL) accounts for speech rhythm, grouping of sounds as either Iambs—if alternating in duration—or Trochees—if alternating in pitch and/or intensity. The two different rhythms signal word order, one of the basic syntactic properties of language. We investigated the extent to which Iambic and Trochaic phrases could be auditorily and visually recognized, when visual stimuli engage lip reading. Our results show both rhythmic patterns were recognized from both, auditory and visual stimuli, suggesting that speech rhythm has a multimodal representation. We further explored whether participants could match Iambic and Trochaic phrases across the two modalities. We found that participants auditorily familiarized with Trochees, but not with Iambs, were more accurate in recognizing visual targets, while participants visually familiarized with Iambs, but not with Trochees, were more accurate in recognizing auditory targets. The latter results suggest an asymmetric processing of speech rhythm: in auditory domain, the changes in either pitch or intensity are better perceived and represented than changes in duration, while in the visual domain the changes in duration are better processed and represented than changes in pitch, raising important questions about domain general and specialized mechanisms for speech rhythm processing.

## Introduction

Spoken language is governed by rhythm and rhythm can be found at almost every single level of speech. At the most basic level, linguistic rhythm is signaled by the time occupied by vowels in the speech stream (V%) and the standard deviation of consonantal intervals (ΔC) (Ramus et al., 1999). Moreover rhythm in spoken language is also signaled through the periodic changes in intensity, duration and pitch involving speech units longer than phonemes such as syllables, which help us to identify for instance which syllables are strong in a word or where is the prominence in phonological phrases. Those changes in intensity, duration and pitch involving phonemes, syllables and other longer linguistic units alternate regularly, conferring to speech prosody a rhythmic alternation.

Because rhythmic alternation at different levels of the rhythmic hierarchy signals different linguistic properties, it offers language learners cues that might allow them to break into different regularities of language detectable from the speech stream. For example, languages can be discriminated on the basis of rhythm at the basic level (e.g., Ramus et al., 1999) and rhythmic alternation offers cues to the size of the syllabic repertoire (Nespor et al., 2011). Continuous speech can be segmented into words (Jusczyk, 1999; Shukla et al., 2007) and phrases (Christophe et al., 1994) on the basis of rhythm, and rhythmic alternation even offers a cue to such basic syntactic properties like word order (Christophe et al., 2003). The ability to represent, recognize and discriminate rhythm in spoken language is therefore likely to play a crucial role for infants acquiring their mother tongue (Langus et al., 2016) and possibly for adults learning a second language. Speech perception, however, is a multi-sensory experience. In addition to the sound of spoken language, speech is also perceived visually from the movements of the lips (McGurk and MacDonald, 1976), the face (Graf et al., 2002; Blossom and Morgan, 2006), the hands (McNeill, 2005; Guellaï et al., 2014), and possibly also from other parts of the body of the speaker. Visual information may be sufficient to discriminate between different languages. For example, bilingual Spanish-Catalan as well as monolingual Spanish and Catalan speakers, but not monolingual speakers of English and Italian, can discriminate Catalan and Spanish using only visual cues (Soto-Faraco et al., 2007). Also monolingual and bilingual English- and Spanish-speaking adults have been shown to discriminate between Spanish and English—two languages differing at the basic rhythmic level—only on the basis of the visual cues provided by speaking faces (Ronquest et al., 2010). These results suggest that adult listeners can discriminate between rhythmically similar (Spanish and Catalan) as well as rhythmically different (English and Spanish) languages by analyzing the facial mimic when they know at least one of the two languages. However, because these visual rhythmic discrimination tasks relied on utterances from languages that differed in prosodic, segmental, lexical and syntactic characteristics, it is difficult to determine which of the speech cues with a visual correlate contributes to the discrimination of the stimuli. Nevertheless, a recent study showed that both, English monolingual and Spanish/Catalan bilingual adults speakers, discriminated resynthesized flat prosody versions of English and Japanese utterances, two languages differing in the mean duration of their consonant and vowel clusters, not only when they were presented as auditory stimuli but also when they were transformed into visual sequences of aperture-close of the mouth of an schematic face, and into vibro-tactile streams (Navarra et al., 2014). Speech rhythm perceived by different sensorial modalities is thus relevant to discover not only segmental but also supra-segmental properties of speech.

Here we therefore investigate audiovisual discrimination of rhythm in phonological phrases in a non-native language. The phonological phrase extends from the left edge of a phrase to the right edge of its head (e.g., the noun in noun phrases or the verb in verb phrases) in head-complement languages; and from the left edge of a head to the left edge of its phrase in complement-head languages (Nespor and Vogel, 1986, 2007). Thus, for example in a language like English a phonological phrase starts with the function word or a preposition and ends with the head, as in for the girls, and in a language like Turkish it starts with the head and it ends at the end of the maximal projection, thus, e.g., a postposition, as in benim için me—for “for me.” Among the languages spoken around the world there are only two known types of phrasal rhythm: iambic and trochaic. Languages with the basic Object-Verb word order, where the head of the phrase follows its complements, such as Turkish and Japanese, mark prominence trochaically mainly through pitch and intensity on the stressed syllable of the first word of the phonological phrase. Languages with the basic Verb-Object word order where the head of the phrase precedes its complements, like English and Italian, mark prominence iambically mainly through duration on the stressed syllable of the last word of phonological phrases (Nespor et al., 2008).

Compared to the other levels of rhythm, phrasal rhythm is likely to play a prominent role in audiovisual discrimination because phrasal prominence is highly salient. The location of prominence at higher levels of the prosodic hierarchy coincides with the location of prominence at lower levels. For example, phonological phrase prominence is signaled on top of lexical stress that is signaled on top of prominence in feet. Thus, prominence at the phonological phrase level is thus acoustically more salient than lexical stress or prominence in feet, which is hardly prominent in connected speech. Furthermore, because phonological phrase boundaries never straddle word boundaries and phonological phrases are fully contained in intonational contours, the rhythm signaled through phonological phrase prominence provides cues to both phrase and word boundaries (Christophe et al., 1994), it signals how words combine into phrases (Nespor and Vogel, 1986, 2007; Langus and Nespor, 2010), and correlates even with the basic word order of the language (Nespor et al., 2008). Sensitivity to phonological phrase rhythm thus provides acoustically highly salient entry points to the understanding of the structure of continuous speech.

Given the importance of rhythm at the phonological phrase level both in language perception and acquisition, could phonological phrase rhythm also be discernable from visual information accompanying speech? At least some prosody is discernable from visual speech cues because the timing and the motor organization of the head are linked to the production of lexical stress, and to prosody in general (Hadar et al., 1983, 1984). When relying only on visual cues of their native language, participants can correctly discriminate the intonation of a statement from that of a question (Srinivasan and Massaro, 2003), detect contrastive focus (Dohen and Loevenbruck, 2005; Dohen et al., 2008), determine when utterances end (Barkhuysen et al., 2008), and identify the location of phrasal as well as lexical stress (Bernstein et al., 1989). In accordance with the fact that prominence at higher levels of the prosodic hierarchy is acoustically more salient, participants are also significantly better at judging the location of phonological phrase prominence than that of lexical stress when relying on visual cues alone (Bernstein et al., 1989).

Adult participants are not only good at perceiving visual prosody: the prosodic cues embedded in the visual information accompanying speech also enhance their perception of speech sounds. For example, speech intelligibility increases when speech is accompanied by nods, by head movements and eye-brows movements (Granström et al., 1999; House et al., 2001; Krahmer et al., 2002; Massaro and Beskow, 2002; Srinivasan and Massaro, 2003; Munhall et al., 2004). Perceivers' judgments about stress are also better with audiovisual speech than with the sound of spoken language alone (Dohen et al., 2008), and the perception of prominence in words is significantly improved when speech sounds are accompanied by hand gestures (Krahmer and Swerts, 2004). Recent findings suggest that prosody in the spontaneous gestures accompanying speech may even help to disambiguate ambiguous sentences (Guellaï et al., 2014). This suggests that adult listeners are quite good at determining the location of phrasal stress in their native language from visual cues alone. However, it remains unclear to what extent adult second language learners are capable of discriminating iambic/trochaic phrasal stress visually in non-native languages.

The ability to discriminate rhythm will depend on the visibility of the main acoustic correlates of phrasal stress: fundamental frequency (F0), duration, and intensity. Because the laryngeal muscles that control fundamental frequency only produce small visible movements in speech production, fundamental frequency is difficult to perceive visually. Even though several studies have tried to associate F0 also with other visual cues that include eyebrows (Cavé et al., 1996) and head movement (Yehia et al., 2002), the evidence for visual perception of F0 is considered impoverished at best (Smith et al., 2010). In contrast, prominence signaled through duration and intensity is realized through the vocal articulators and is therefore considerably more visible than fundamental frequency. The pronunciation of stress is in fact associated with larger, faster and longer jaw and lip movements (e.g., Beckman and Edwards, 1994; de Jong, 1995; Erickson et al., 1998; Harrington et al., 2000; Erickson, 2002; Cho, 2005, 2006). The role of the articulators in stress perception is also supported by the findings that judgments about phrasal stress are not affected when the face is hidden from the nose up, suggesting that the mouth may contain enough information for discriminating trochaic phrasal stress signaled through intensity and iambic phrasal stress signaled through duration (Lansing and McConkie, 1999). Perceiving prominence signaled through pitch—that is difficult to discern visually—should therefore rely more on stable auditory information. In contrast, perceiving prominence signaled through intensity and duration, both of which are visible in the face of the speaker, could also rely on visual information.

This raises the issue of the role of audio-visual information in speech perception. While auditory and visual information clearly contribute to the perception of supra-segmental information, the differences in the visibility of the different acoustic correlates of prominence also suggest that they are likely to contribute differently to speech perception. The majority of studies that investigate the audio-visual perception of prosody test how auditory and visual information are integrated in speech perception. This generally entails comparing participants' performance either exclusively in the auditory modality or exclusively in the visual modality to participants' performance with audio-visual speech. However, because auditory information alone is often sufficient for participants to perform at ceiling (e.g., Brunellière et al., 2013), the importance of visual cues in speech perception has remained difficult to describe. Rather than testing the advantages of audio-visual rhythm over rhythm perceived from the single modalities, we tested participants' ability to discriminate iambic/trochaic rhythm within and across the two modalities. By comparing participants' performance in discriminating iambic/trochaic rhythm either in the auditory modality alone (Experiment 1a), or in the visual modality alone (Experiment 1b), we will investigate whether there are significant differences between auditory and visual cues in phonological phrase rhythm. By testing whether participants can transfer rhythm from the auditory to the visual modality (Experiment 2a) and vice versa (Experiment 2b), we aim at discovering whether participants can dynamically integrate information from the auditory modality with information from the visual modality, when such information is simultaneously only available from a single source (either audio or visual). For example, can participants switch from auditory to visual prosody (or vice versa) when the first of the two becomes degraded or inaudible due to situational constraints?

In this paper, we therefore investigate adult participants' ability to match rhythmic patterns in audio and visual presentations of faces uttering either iambic or trochaic nonsense phrases. Although recognition of Iambs and Trochees has been extensively investigated auditorily (Hay and Diehl, 2007; Iversen et al., 2008; Bion et al., 2011; Bhatara et al., 2013), the evidence for Iambic-Trochaic Law (ITL) in the visual domain is highly scarce. To the best of our knowledge in the visual domain only one previous study has shown that visual sequence of colored squares are grouped respecting the ITL (Peña et al., 2011). To the best of our knowledge, no study has reported that iambic/trochaic rhythm can be recognized from the visual information provided by lips and mouth when perceiving non-native languages; neither that the information obtained from ITL in one modality, i.e., auditory or visual, can be transferred to the other sensorial modality. Since the ITL is informative about word order, and visual and auditory cues are exploited for language learning, our study will add new data on how particular audio-visual cues might support language processing and learning. We recorded the stimuli from German native speakers because in German, subordinate clauses, depending on the type of complementizer chosen, can either have the Object-Verb order (e.g., *weil ich papa sehe* translated in English as *because I father see*), where phonological phrases prominence is signaled trochaically at the beginning of the phrase, or the Verb-Object order (e.g., *denn ich sehe papa* translated in English as *because I see father*) where phonological phrases prominence is signaled iambically at the end of the phrase (Nespor et al., 2008). We replaced the object-verb (e.g., *papa sehe*)/verb-object (e.g., *sehe papa*) pairs in these constructions with nonsense words and then video recorded German native speakers uttering the resulting nonsense subordinate clause. These Object-Verb/Verb-Object constructions within a single language enabled us to test the discrimination of iambic-trochaic rhythm in a controlled way. That is, the discrimination between iambic-trochaic phonological phrase prominence could not occur due to differences caused by cross-linguistic and cross-speaker differences. In addition, the rhythmic differences could only be related to phonological phrase prominence rather than to either lexical stress or secondary stress within feet, since the latter are identical in the two types of phrases. In addition all relevant phrases were uttered in a non-emphatic way—as if out of the blue—and were included in a single intonational phrase. Thus, the rhythmic differences could not be caused by different intonational phrase breaks.

## Experiment 1: matching iambic and trochaic items from unimodal contexts

Even though the ITL has been investigated in spoken language, as well as in tones (e.g., Hay and Diehl, 2007) and in visual stimuli (Peña et al., 2011) we know of no study that directly compares adult participants' ability to discriminate phrasal prominence in both the auditory and the visual component of speech samples. In Experiment 1a we therefore tested Spanish-speaking adult listeners' ability to discriminate iambic and trochaic phrasal prominence within auditory stimuli that were recorded from German speakers in whose native language phrasal prominence in subordinate clauses can be either iambic or trochaic. There is no experimental evidence that shows that speakers of Spanish, an iambic language where phrasal prominence is signaled primarily through duration at the end of phonological phrases, can recognize Iambs and Trochees in linguistic stimuli. However, on the basis of previous findings from speakers of other iambic languages such as Italian and English, we predict that also Spanish speakers will discriminate Iambs from Trochees. Spanish speakers are also likely to succeed in this task because violations in perceiving iambs have only been found in trochaic languages, that have a basic Object-Verb word order (e.g., Turkish and Persian), but never in iambic languages (e.g., Italian and English), that have a basic Verb-Object order (Langus et al., 2016). On the one hand, testing Spanish-speakers with German stimuli would thus provide evidence for the perception of iambs and trochees at the phrasal level in a language that has previously not been investigated.

Following the discrimination in the auditory modality, in Experiment 1b, we tested Spanish-speaking adult listeners' ability to discriminate iambic and trochaic phrasal prominence within visual stimuli that were extracted from the same audio-visual recordings used in Experiment 1a. Previous findings with visual speech suggest that adult participants are highly accurate in determining the location of phrasal prominence in visual presentations of their native language (Soto-Faraco et al., 2007). Is it possible that hearing adults could also discriminate Iambic and Trochaic nonsense phrases extracted from the visual prosody of a foreign language? On the one hand, the iambic and trochaic prominences in phonological phrases differ not only in location but also in the acoustic correlates that signal it: pitch and intensity at the beginning of the phrase in the case of trochaic rhythm and duration at the end of the phrase in the case of iambic rhythm. This suggests that participants have at least two cues - prominence location and the acoustic correlate of prominence—for discriminating between iambic and trochaic phrases. However, it is unknown whether these cues are available when perceiving foreign speech visually. We thus expect that Spanish-speaking adults would detect the prosodic properties involving pitch and duration in the auditory presentations of trochaic and iambic nonsense phrases, respectively (Experiment 1a), as well as in the visual representation of pitch and duration in the movements of the mouth and the lips (Experiment 1b).

## Experiment 1a: matching iambic and trochaic items in the auditory modality

We tested whether Spanish-speaking adults can discriminate iambic and trochaic nonsense phrases extracted from German prosody when only auditory cues are available.

### Materials and methods

#### Participants

Eighteen college students (9 male, 9 female; mean age = 26.5 years) completed the study. In this, as well as in all experiments of our study, participants were native speakers of Spanish with either normal or corrected to normal vision. They received academic credits for their participation, and, as in all experiments of this study, signed a written consent form approved by the local ethical committee.

#### Stimuli

We video-recorded two native speakers of German (one female and one male, both 25 years old) while they uttered a series of 6 nonsense phrases (“bole tase,” “bale tose,” “dofe mave,” “dafe move,” “move fape,” “mave fope”). In order to record the same nonsense phrases with natural iambic and trochaic prosody in a single language, we chose to record native speakers of German because it is a language where both forms are equally used (Nespor et al., 2008). We prepared a written list of nonsense phrases by replacing the last two words of an iambic and a trochaic real phrase. In the real phrase, e.g., *denn ich sehe papa* (“because I see father”), that has an iambic structure, we replaced *sehe papa* by each one of the six nonsense two-words phrases, e.g., *denn ich bole tase*, and in the real phrase *weil ich papa sehe* (“because I see father”), that has a trochaic structure, we replaced *papa sehe* by the same nonsense phrases, e.g., *weil ich bole tase*. Speakers were unaware of the purpose of the experiment and were asked to read the list and utter each sentence with the same prosody they use for uttering the real sentence. Each sentence was randomly presented 6 times in the list. To avoid participants' tendency to direct their gaze to the speakers' eyes, we asked speakers to wear black sunglasses. Speakers were also asked to avoid facial mimic and to talk in non-emphatic speech. In a second step, we segmented the last two nonsense words of each video recording, and selected the best 5 iambic and 5 trochaic exemplars (hereafter nonsense phrases) from each speaker. We chose sequences that had similar duration and pitch, and did not contain salient head movements or facial mimic. The resulting stimuli were prepared as auditory and visual files, containing either only the auditory or only the visual track of the nonsense phrases (see Supplemental Videos and sounds). In Experiment 1a only auditory files were used.

To quantify whether and how the stimuli acoustically differed in pitch distribution and duration, for each speaker we first measured the duration and the maximum F0 for of each one of the four syllables of each phrase, and we then statistically compared these values in trochees vs. iambs. We did verified that the maximum F0 in the first syllable was significantly higher for Trochaic than for Iambic stimuli, and that the duration of the third syllable was significantly larger for Iambs than for Trochees in both female and male speakers. Moreover, only in female speaker, the pitch of the second syllable was also significantly higher for trochees than for iambs (see Table 1).

**Table 1:** **Duration and pitch of the auditory tracks of iambs and trochees stimuli**.

		**Duration**		**Pitch**	
		**Female**	**Male**	**Female**	**Male**
Syllable 1	Iamb	238 ± 46	214 ± 31	280 ± 12	136 ± 8
	Trochee	239 ± 43	229 ± 19	338 ± 13	152 ± 10
	p	n.s	n.s	<0.001	<0.001
Syllable 2	Iamb	153 ± 27	135 ± 18	296 ± 12	139 ± 6
	Trochee	155 ± 28	138 ± 18	326 ± 27	131 ± 18
	p	n.s.	n.s.	<0.001	n.s.
Syllable 3	Iamb	368 ± 41	299 ± 46	275 ± 24	124 ± 13
	Trochee	321 ± 67	253 ± 69	265 ± 19	118 ± 5
	p	0.006	0.019	n.s.	n.s.
Syllable 4	Iamb	366 ± 25	274 ± 41	238 ± 10	117 ± 9
	Trochee	360 ± 32	271 ± 38	247 ± 17	115 ± 6
	p	n.s.	n.s.	n.s.	n.s.

We indicate the mean and standard deviation of duration and maximum F0 over each of the four syllables of each iambic and trochaic stimuli, for the female and for the male speaker. For each speaker, we submitted the duration and maximum F0 measured over each syllable in each one of the iambic and trochaic stimulus, to two separated paired t-test (alpha 0.05, 2 tails), one t-test for duration and one for F0. The p-values for those comparisons are indicated per syllable and speaker.

#### Trial structure

Participants heard 48 randomly presented trials, 12 in each of the four experimental conditions that resulted from the combination of two trial types, (i.e., without a change, hereafter standard trial and with a change, hereafter deviant trial) and two prosodic contrast types (i.e., iambic and trochaic). Each trial comprised the presentation of four consecutive nonsense phrases uttered by a single speaker, separated by 1000 ms of no stimulation. Inter-trial interval varied from 1500 to 3000 ms.

#### Procedure

**Figure 1:**
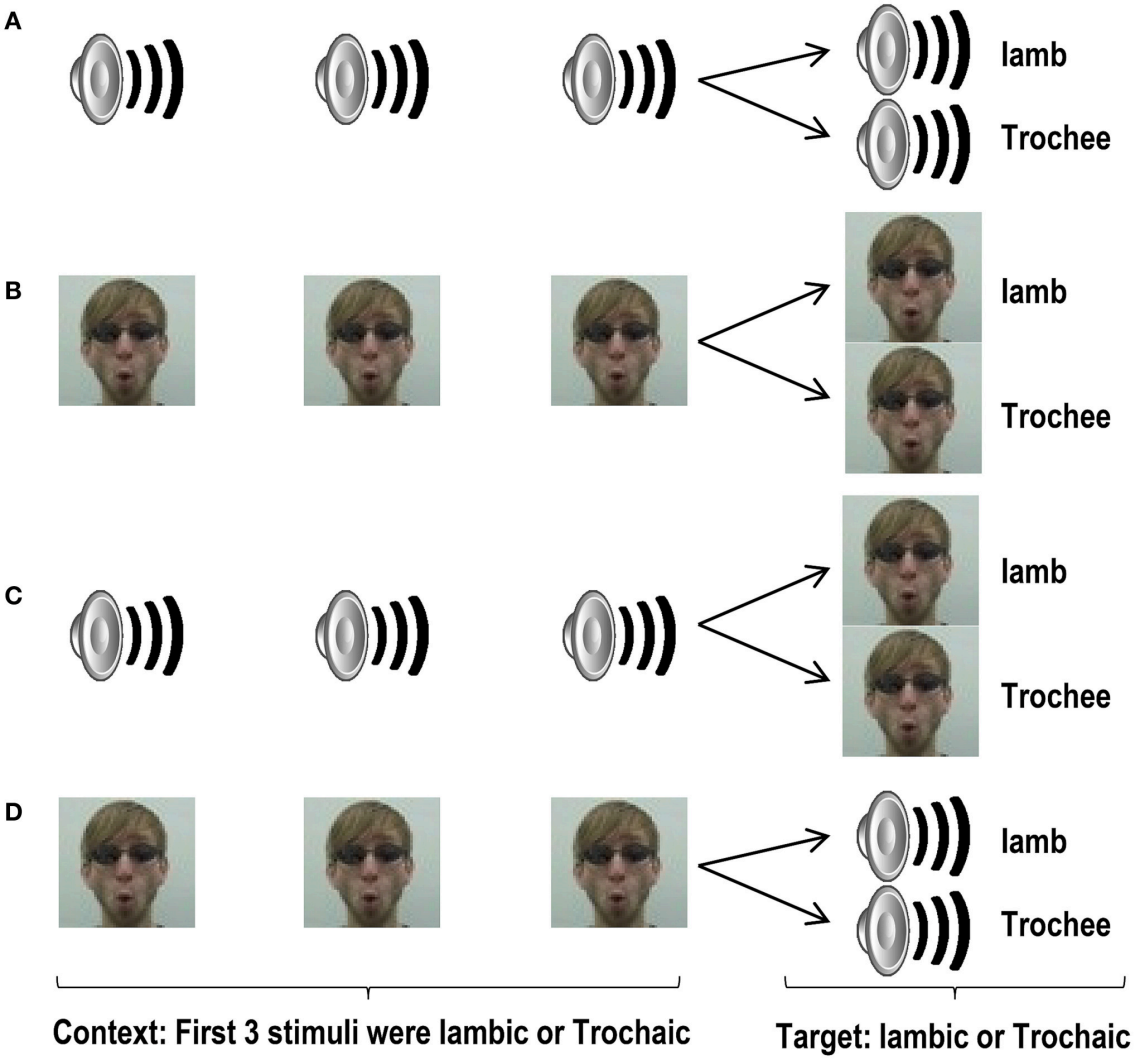
**Schematic structure of trials in Experiment 1a (A)**, 1b **(B)**, 2a **(C)**, and 2b **(D)**. Loudspeaker and face still images represent auditory and visual video files, respectively. The first 3 stimuli in each trial (the context), and the last stimulus of each trial (the target) were different exemplars of a prosodic category. However, the 4th stimuli were identical across standard and deviant trials.

All experiments were carried out in a dimly lit soundproof room (0.05–0.1 μW/cm^2^). Participants were seated at 60–70 cm distance from the screen and heard the stimuli through headphones. Written instructions were presented on a computer screen. Before starting the experiment each participant was informed that in normal speech people can utter a single phrase in different ways, e.g., *welcome*! vs. *welcome*?, and that this study explored their ability to recognize similarities and differences in the prosody of nonsense phrases, by listening to them. We emphasized that similarities and differences will only involve prosody, and we gave them one practice trial of each trial type using material not included in the experiment. Participants were then informed that in all trials, the first three stimuli were three different exemplars of a same nonsense phrase pronounced with the same melody, e.g., *welcome*!—*welcome*!—*welcome*!. In contrast, in half of the trials the fourth nonsense phrase will be uttered with the same melody, such as *welcome*! in the previous example, while in the other half, it will be uttered with a different melody, e.g., *welcome*?. We thus exposed participants to trials with no change in prosodic contrast where all the four exemplars matched, i.e., iambic1- iambic2- iambic3- iambic4, or trochee1- trochee2- trochee3- trochee4 (i. standard trials), and to trials with change in prosodic contrast where the fourth exemplar mismatched the previous three exemplars, i.e., iambic1- iambic2- iambic3- trochee4, or trochee1- trochee2- trochee3- iamb4 (i.e., deviant trials). Participants were asked to attentively listen to the four nonsense phrases of the trials because at the end of each trial they would be asked to judge if the fourth nonsense phrase had the same or a different prosody from the three preceding phrases. They were asked to respond as accurately and as fast as possible by pressing the “same” or the “different” button. Figure 1A illustrates the structure of the trials in this experiment.

#### Data analysis

Data analysis was similar for all the experiments. For each participant in each prosodic contrast type (i.e., iambic and trochaic) and each trial type (i.e., standard and deviant) we measured the mean accuracy as the percentage of correct responses (i.e., percentage of correct Same + percentage of correct Different over all trials), and the mean reaction time for correct responses. To estimate participants' perceptual discriminability and response bias to the prosodic contrasts and to determine whether there was a bias for participants in our “same-different” task, we computed *A*–prime (hereafter *A'*) and *Beta* (hereafter *B*”), respectively. A' measures participants ability to discriminate iambs from trochees in the given task estimating the probability to answer “same” when the target was the same and to answer “different” when the target was not the same. B” measures the bias that participants may have to answer “same” or “different” regardless of target. A' 0.5 means performance at chance, near to 1.0 indicates good discriminability. B” equal to 0.0 indicates no bias, while positive and negative numbers (until a maximum of −1 and 1) reflect a tendency to answer “different” and “same,” respectively.

For the statistical analysis, we first computed *A'* and *B”* for each participant by using classical algorithms (Snodgrass et al., 1985; Mueller and Weidemann, 2008). We then submitted A' of all participants to a one sample *t*-test (alpha = 0.05; two-tailed) compared against chance (i.e., 0.5), and we computed the mean B” for the group. Subsequently, only if *A'* was significantly different from chance for each group, that is, if data showed good discriminability, we submitted the mean accuracy of participants for Iambic and Trochaic trials to separated one sample *t*-test (alpha = 0.05; two tailed) against chance (50%). Moreover, to estimate possible differences in the ability to recognize Iambic and Trochaic items, we submitted the mean accuracy and the mean reaction time of participants for both types of Prosodic contrast to a repeated measure ANOVA with Prosodic contrast (Iamb and Trochee) as within-subject factor with Greenhouse-Geisser correction.

## Results

**Figure 2:**
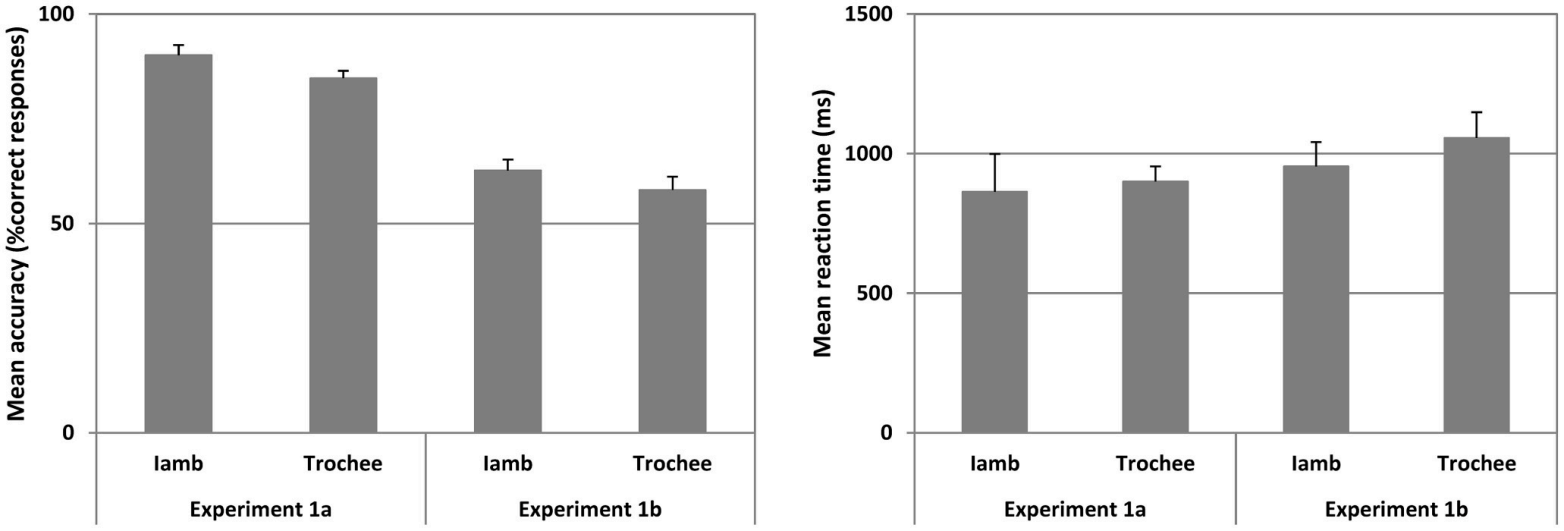
**Mean accuracy (left panel) and mean reaction time (right panel) for Experiments 1a and 1b for Iambic and Trochaic trails are plotted**. Fifty percent accuracy represents the chance level. Error bars indicate 95% confidence interval.

The mean accuracy and mean reaction times for correct responses in Iambic and Trochaic trails are illustrated in Figure 2.

The mean *A'* across all trials was significantly higher than chance [Mean = 0.927 ± 0.045, *t*_(17)_ = 39.920, *p* < 0.001, Cohen's *d* = 13.81] showing that subjects did discriminate the phonological cues evaluated in this study. Moreover, the mean B” was 0.060 (range = 0.584 to −0.464), showing that the group of participants did not show a tendency to press either mostly the “Same” or mostly the “Different” key in this study. We thus submitted the mean accuracy for Iambic and Trochaic trials to a separated one-sample *t*-test (alpha = 0.05; two-tailed) compared against chance (50%). Mean accuracy was significantly above chance for Iambic [Mean ± SD = 90.135 ± 10.374, *t*_(17)_ = 16.413, *p* < 0.001, Cohen's *d* = 5.61], and Trochaic trials [Mean ± SD = 84.670 ± 7.486, *t*_(17)_ = 19.650, *p* < 0.001, Cohen's *d* = 6.74]. No significant differences were observed in statistical comparisons of mean accuracy for Iambs and Trochees, or participants' reaction times for correct responses across conditions. Our results thus show that both Iambic and Trochaic nonsense phrases are recognized by adult Spanish speakers in the auditory modality.

Due to previous studies that suggest that facial mimic, particularly eyebrow, may serve as a cue associated to pitch increase, we explored this possibility. We classified the trials in with and without salient eyebrow movement, regardless if they were iambics or trochaics, in each speaker. We found that the male speaker frequently elevated the eyebrow especially when uttered trochees. In contrast, the female speaker rarely elevated her eyebrows. We then measured the accuracy for iambs and trochees separately in trials with and without eyebrow movements for iambic and trochaic (match and mismatch). In both types of trials we found similar results than those observed when we compared all trials together, suggesting that eyebrow is a salient cue but not indispensable to make the task.

## Experiment 1b: matching iambic and trochaic items in the visual modality

We tested whether adult Spanish-speaking adults can discriminate iambic and trochaic nonsense phrases extracted from German prosody when only visual cues are available. The results of Experiment 1a are directly comparable to the results of Experiment 1b because the visual stimuli of Experiment 1b are extracted from exactly the same audio-visual recordings as the auditory stimuli of Experiment 1a.

### Method

#### Participants

Eighteen college students (7 male, 11 female; mean age = 21.5 years) completed the study. None of the participants participated in Experiment 1a.

#### Stimuli

The stimuli corresponded to the visual track (mouth and lip movement plus facial mimic associated to speech) of the nonsense phrases evaluated in Experiment 1a, where the auditory information was completely removed.

#### Procedure

The procedure was similar to that described for Experiment 1a, but this time the stimuli were presented exclusively as videos. Participants received instructions similar to those of Experiment 1a, but were informed that the goal of this study was to explore whether the different ways to utter a single phrase can be visually detected by exploring how the lips and the mouth of the speaker move. Before starting the experiment participants saw an example of each trial type. Participants were then asked to attentively watch the lips/mouth of the speakers because at the end of each trial they would have to judge if the fourth video used the same or a different prosody of the three preceding videos. Responses were given by pressing the “same” or “different” button. Figure 1B illustrates the structure of the trials.

### Results

**Figure 3:**
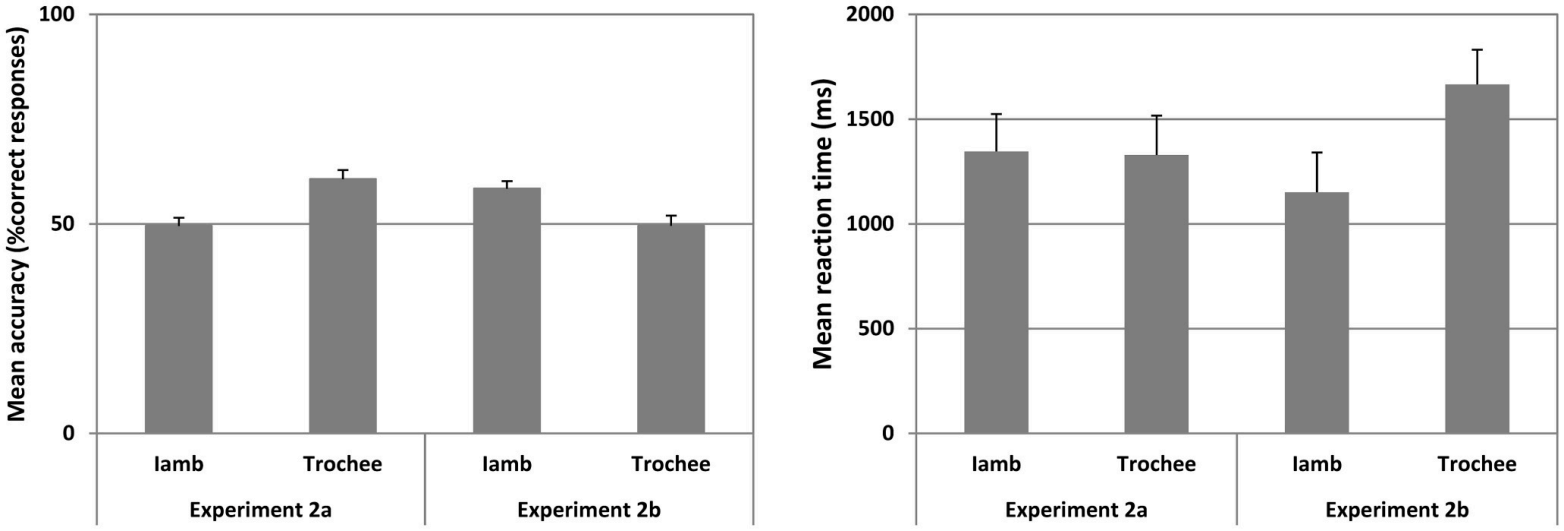
**Mean accuracy (left panel) and mean reaction time (right panel) for Experiment 2a and 2b for Iambic and Trochaic trails are plotted**. Fifty percent accuracy represents the chance level. Error bars indicate 95% confidence interval.

The mean accuracy and mean reaction time for the correct responses in Iambic and Trochaic trails are illustrated in Figure 3. We found that the mean B” was −0.507 (range = −0.258 to −0.765), suggesting that the group of participants did have a tendency to press “same” in this Experiment. However, the mean A' across all trials was significantly higher than chance [Mean = 0.678 ± 0.104, *t*_(17)_ = 7.203, *p* < 0.001, Cohen's *d* = 2.49], showing that despite the slight tendency to press “same” participants were highly able to discriminate each one of the prosodic contrast we were testing. We thus submitted the mean accuracy for Iambic and Trochaic trials to separated one-sample *t*-test (alpha = 0.05; two-tailed) compared against chance (50%). We found that mean accuracy was significantly above chance for both Iambic [Mean ± SD = 62.611 ± 11.231, *t*_(17)_ = 4.629, *p* < 0.001, Cohen's *d* = 1.63] and Trochaic trials [Mean ± SD = 57.975 ± 13.548, *t*_(17)_ = 2.498, *p* = 0.023, Cohen's *d* = 0.86]. No other significant differences were observed. Results show that participants discriminate the presence and absence of changes in both the duration (Iambic) and the pitch (Trochaic) of the target. Compared to Experiment 1a, our results suggest that visual cues underpinning duration and pitch are detectable from faces by normally hearing persons.

To quantify the differences between the results of Experiment 1a and those of Experiment 1b, we submitted the results of both experiments to a *t*-test for two independent samples (alpha = 0.05, two-tailed). We found that the accuracy in Experiment 1b was significantly lower than that observed in Experiment 1a for both Iambic (*p* < 0.001) and Trochaic (*p* < 0.001) trials, showing that recognizing prosody from visual cues is a harder task than from auditory cues. No significant differences were found in reaction time.

## Experiment 2: matching iambic and trochaic targets from a context with different modality

The results of Experiments 1a and 1b show that Spanish-speaking adults can distinguish iambic-trochaic patterns in both the auditory and the visual modality of a foreign language. This suggests that the representations of phrasal rhythm—i.e. whether prominence is signaled trochaically at the beginning of the phrase or iambically at the end of the phrase—are not modality specific. However, if the representations extracted within the auditory and visual modalities emerge from amodal or multimodal processing, participants should also be able to use the information extracted in one modality to detect iambic-trochaic patterns in the other modality, even when the audio and visual components of speech are not presented simultaneously. In the next two experiments, we therefore explored whether participants can discriminate iambic phrasal prominence presented in one modality from trochaic phrasal prominence presented in another modality and vice versa. If the representation of phrasal rhythm is not modality specific we would predict that adult Spanish-speaking participants' performance in discriminating iambic-trochaic rhythm across the audio-visual modalities would parallel their performance in Experiments 1a and 1b. In Experiment 2a, we therefore tested whether participants can discriminate visual iambic or trochaic targets when they were familiarized with auditory examples, and in Experiment 2b, whether they could discriminate auditory iambic or trochaic phrases when familiarized with visual examples. The ability to correctly discriminate rhythm from one modality to another would constitute a strong evidence for the multimodal representations of rhythm in audiovisual speech perception.

## Experiment 2a: matching visual iambic and trochaic targets from auditory context

We tested whether Spanish-speaking adults can discriminate iambic and trochaic nonsense phrases extracted from German prosody when the context of each trial is presented only auditorily and the test items are presented only visually.

### Method

#### Participants

Eighteen college students (8 male, 10 female; mean age = 23.5 years) completed the study. None of the participants had participated in the previous experiments.

#### Stimuli

The stimuli were the auditory and visual tracks of the nonsense phrases evaluated in Experiment 1a.

#### Procedure

Procedure and instructions were similar to those of Experiments 1a and 1b, but this time participants were informed that the first three stimuli of each trial would be presented auditorily, while the target will appear visually. Participants were shown an example of each trial type and were asked to attentively listen and watch the lip/mouth of the speakers because at the end of each trial they would have to judge if in the fourth video the speaker used the same or a different prosody as that used in the three precedent audio files. Responses were given by pressing the “same” or the “different” button. Figure 1C illustrates the structure of the trials.

### Results

The mean accuracy and mean reaction time for correct responses in Iambic and Trochaic trials are illustrated in Figure 3. The mean *B”* was −0.415 (range = −0.080 to −0.750), suggesting that the group of participants did have a tendency to press “Same” in this Experiment. However, the mean *A'* across all trials was significantly higher than chance [Mean = 0.618 ± 0.074, *t*_(17)_ = 6.737, *p* < 0.001, Cohen's *d* = 2.32], showing that despite the tendency to press “same” participants were able to discriminate the prosodic contrast we were testing. We thus submitted the mean accuracy for Iambic and Trochaic trials to separated one-sample *t*-test (alpha = 0.05; two-tailed) compared against chance (50%). We found that mean accuracy was significantly above chance only for Trochaic trials [Mean = 60.685 ± 9.375, *t*_(17)_ = 4.709, *p* < 0.001, Cohen's *d* = 1.66]. Moreover, mean accuracy in Trochaic trials was significantly higher than that observed in Iambic trials [*F*_(1, 17)_ = 17.270, *p* = 0.001, eta2p = 0.504]. No significant differences in reaction time were observed. Results show that only when the auditory context is Trochaic, i.e. it had phrases with higher pitch in the initial word, participants were able to match similarities and discriminate variations in the visual target. Our results thus only support our prediction that when the auditory familiarization involves trochees, match and mismatch of visual targets would be better than chance. This suggests that the transference of ITL properties from an auditory context to a visual format is harder when the auditory familiarization involves duration cues as compared to when it concerns cues related to pitch. Traces of memory for pitch appear to trigger more stable representations during familiarization than traces of memory for duration, allowing a better performance in both the match and the mismatch of the visual target relative to auditory familiarization. Previous research on infants is in agreement with our data showing that pitch and duration in speech are asymmetrically processed. While subtle changes in pitch are quite easily detected by infants at 7 months of age, proportionally similar changes in duration are not (Bion et al., 2011).

## Experiment 2b: matching auditory iambic and trochaic targets from visual context

In this experiment, we tested whether Spanish-speaking adults can discriminate iambic and trochaic nonsense phrases extracted from German prosody when the context of each trial is presented only visually and the test items are presented only auditorily.

### Method

#### Participants

Eighteen college students (11 male, 7 female; mean age = 20.8 years) completed the study. None of the participants had taken part in the previous experiments.

#### Stimuli

The stimuli were the same audio and visual track of the nonsense phrases evaluated in Experiment 2a.

#### Procedure

Procedure and instructions were similar to those of Experiment 2a, but this time we informed participants that the first 3 stimuli of each trial would be presented visually while the target would be presented auditorily. Participants were shown an example of each trial type and were asked to watch attentively the lip/mouth and listen to the speakers because at the end of each trial they would have to judge if in the fourth audio file the speaker used the same or a different prosody as s/he used in the three preceding videos. Responses were given by pressing “same” or “different” button. Figure 1D illustrates the structure of the trials.

### Results

The mean accuracy and mean reaction time for correct responses in Iambic and Trochaic trails are illustrated in Figure 3. The mean B” was −0.452 (range = −0.083 to −0.821), suggesting that the group of participants did have a tendency to press “Same” in this Experiment. However, the mean A' across all trials was significantly higher than chance [Mean = 0.620 ± 0.060, *t*_(17)_ = 6.384, *p* < 0.001, Cohen's *d* = 2.91), showing that despite the tendency to press “Same,” participants were able to discriminate the prosodic contrast we were testing. We thus submitted the mean accuracy for Iambic and Trochaic trials to separated one-sample *t*-test (alpha = 0.05; two-tailed) compared against chance (50%). We found that mean accuracy was significantly above chance only for Iambic trials [Mean ± SD = 58.161 ± 7.765, *t*_(17)_ = 2.868, *p* < 0.010, Cohen's *d* = 1.53]. No significant differences were observed in accuracy between Iambic and Trochaic trials, although the mean for Trochees was near chance. Similarly no difference was found in reaction times. The results show that participants discriminate the presence and absence of changes in the target only when the context was visual iambic, suggesting that duration cues allow the emergence of more stable multimodal linguistic representations during visual familiarization. Visual iambic stimuli can thus be matched and mismatched to the auditory modality with higher performance than visual trochees. To estimate the differences observed in Experiments 2a and 2b we submitted the mean accuracy in both experiments to a repeated measures ANOVA with prosodic contrast (Iamb and Trochee) as within-subject factor, and Experiments (2a and 2b) as between-subject factor with Greenhouse-Geisser correction. We found a significant interaction Prosodic Contrast X Experiment [*F*_(1, 32)_ = 10,932; *p* = 0.002, eta2p = 0.255], due to the fact that the mean accuracy for Trochees was significantly higher in Experiment 2a than in Experiment 2b [*F*_(1, 32)_ = 8832, *p* = 0.006, eta2p = 0.216], and the mean accuracy for Iambs was significantly higher in Experiment 2b than in Experiment 2a [*F*_(1, 32)_ = 4.194, *p* = 0.049, eta2p = 0.116]. Our results thus demonstrate participants' difficulty to match audio correlates of ITL to the visual correlate of ITL when familiarization and target stimuli were presented consecutively. This may mean that speech perception is fastest when the auditory and visual components of speech are perceived simultaneously (e.g., Soto-Faraco et al., 2007).

## General discussion

Our results show that Spanish-speaking adults can discriminate Iambic and Trochaic phrasal rhythms both in the auditory and in the visual modality even when the stimuli have prosodic patterns that do not match those that are unmarked in their native language, such as trochees for Spanish speakers. Because changes in duration and intensity involve changes in the lips and the anterior mouth configuration, these correlates are highly salient in the visual modality (Smith et al., 2010). Pitch, however, being generated by the frequency of vibration of the vocal cords, is considerably less salient in the visual modality and has proven difficult to be discerned from the lips, mouth and other movements associated to speech facial mimic (Vatikiotis-Bateson and Yehia, 1996), although eyebrows may be a strong cue to reflect this parameter in hearing persons (Borràs-Comes and Prieto, 2011; Prieto et al., 2015). Phrasal rhythm can either be signaled mainly through duration (iambic) or mainly through pitch and intensity (trochaic). Spanish-speaking participants' ability to discriminate phrasal rhythm visually thus suggests that participants are sensitive to the visual correlates of duration and pitch/intensity patterns from a foreign language. Furthermore, because the visual displays of our stimuli covered the eyes of the speaker, our results suggest that suprasegmental linguistic rhythm is discernable also solely from the movements of the mouth and the lips involved in speech production.

Our current results extend previous findings on audio-visual discrimination of speech through visual cues only. For example, it has been found that both bilingual and monolingual Spanish and Catalan speakers, but not speakers of English and Italian, can discriminate Catalan and Spanish using only visual cues (Soto-Faraco et al., 2007). Also both monolingual and bilingual English- and Spanish-speaking adults have been shown to discriminate between Spanish and English only on the basis visual cues (Ronquest et al., 2010). Likewise, Navarra et al. (2014) showed that English and Spanish/Catalan adult speakers do exploit visual information concerning the temporal distribution of consonant and vowel intervals to discriminate languages that differ in this speech property such as English and Japanese. These results suggest that adult listeners can discriminate between rhythmically similar (Spanish and Catalan) as well as rhythmically different (English and Spanish) languages when they know at least one of the languages. In most languages, auditory phrasal prominence can be either iambic (e.g., in Italian, French and English) or trochaic (e.g., in Turkish, Japanese, and Persian). Our results show that Spanish-speaking adults can discriminate between iambic/trochaic phrasal rhythm using only visual cues. Importantly, because the stimuli of our experiments were recorded by German speakers and modeled on the iambic/trochaic subordinate clauses in German, and our participants were native speakers of Spanish, our results also support the view that language discrimination using only visual cues does not necessarily require knowledge of the target language. Because Spanish is an iambic language that signals phrasal prominence with duration at the end of phrases, our results with German stimuli also suggest that speakers of iambic languages are capable of discriminating between iambic and trochaic phrasal rhythms, when only visual cues are available. Our results cannot directly attest whether also speakers of trochaic languages can discriminate iambic and trochaic phrasal rhythm in a similar manner. Previous findings with trochaic languages, such as Turkish and Persian, suggest that listeners violate the ITL by grouping syllables alternating in duration as well as pitch trochaically (Langus et al., 2016). However, because in a discrimination task participants do not necessarily have to group syllables into phrases, and they simultaneously rely on the location of the phonological phrase prominence (initial/final) as well as on the specific acoustic/visual correlate of prominence (pitch/duration/intensity), it is likely that also speakers of trochaic languages would be able to discriminate between two unknown languages differing in phrasal prominence.

Despite participants' high accuracy in discriminating phrasal prominence within the auditory and visual modalities, it is important to note that there were significant differences between participants' accuracy in matching prosodic contrasts across modalities. In fact, there is no perfect transfer of rhythm between the auditory and the visual modalities when they are presented consecutively. Participants in our experiments could only match iambic patterns from the visual to the auditory speech modality, and trochaic patterns from the auditory to the visual speech modality. While the representations of Iambs acquired through the visual perception of speech appear to be multimodal—i.e., can be transferred to visual as well as to auditory targets—they are modality specific when perceived in the auditory modality—i.e., they can only be transferred to the auditory modality. Exactly the reverse is true for trochees that are multimodal when acquired in the auditory modality but modality specific when perceived in the visual modality. The fact that participants appear to be highly consistent in auditory-to-visual transfer (trochees) and in visual-to-auditory transfer (iambs) suggests that these differences emerge due to the representations—rather than due to general limitations of transfer—of rhythm in the auditory and visual modalities of speech.

Our results thus suggest that audiovisual speech perception is complementary: the information that is transferred from the auditory to the visual domain does not also need to be transferred from the visual to the auditory domain. At least for the audiovisual perception of phrasal rhythm, the processing of spoken language has found an ecological way to distribute the amount of information that must be transferred. This is problematic for theories that see audiovisual speech perception as superior to speech perception only in the auditory domain (Sumby and Pollack, 1954; Hardison, 2003). Our results suggest that perceiving speech audio-visually in adverse conditions is not always beneficial. Because the transfer of information depends on the direction of transfer (auditory-to-visual or visual-to-auditory) listeners will benefit differently when perceiving speech in a noisy environment or when perceiving speech when the face is not clearly visible (Sumby and Pollack, 1954; Hardison, 2003). Thus, while in perfect conditions of audio-visual speech perception both auditory and visual cues complement each other to better understand speech, their usefulness in adverse conditions may differ considerably.

While it has been shown that facial mimic (Weikum et al., 2007) and lip reading (Bristow et al., 2009; Yeung and Werker, 2013) are cues for language and phoneme discrimination, respectively, our results show that also the prosodic information carried by the visual component of speech is detectable from the speaker's lips. It is of course possible that pitch might be detected visually more clearly from the eyebrows (Krahmer and Swerts, 2004), as is the case in sign languages (Nespor and Sandler, 1999). Further research is needed to investigate the range of pitch information that can be detected in speech from the eyebrows. The extent to which information from the auditory and the visual modalities can be combined to aid the perception and comprehension of the prosody of human language will depend on how accessible the different aspects of language in the specific modalities are.

Our results show that hearing persons exploit the ITL to process speech in both the auditory and the visual modality, supporting once more a general mechanism responsible for grouping as predicted by the ITL.

## Author contributions

MP, MN, and AL designed the study, CG and DH acquired the data, AL and MP analyzed the data, MP, MN, and AL interpreted the data and wrote the manuscript. MP, AL, CG, DH, and MN approved the final version of the manuscript to be published and agreed to be accountable for all aspects of the work in ensuring that questions related to the accuracy or integrity of any part of the work are appropriately investigated and resolved.

### Conflict of interest statement

The authors declare that the research was conducted in the absence of any commercial or financial relationships that could be construed as a potential conflict of interest.
