# Supplementary material for: Rhythm on Your Lips
Source: Front Psychol. 2016 Nov 8;7:1708. doi: 10.3389/fpsyg.2016.01708 (PMC5099249; doi:10.3389/fpsyg.2016.01708)
Supplement: Supplementary file 1 [file Table1.PDF]

Supplemental Materials:

An example of audio and visual stimuli used in this study are included.

audioIamb.mov contains an example of the sound track of an iambic non-sense phrase. Digitalized at 16 bit Integer (Big Endian), 22050 Hz, Mono.

audioTrochee.mov contains an example of the sound track of a trochaic non-sense phrase. Digitalized at 16 bit Integer (Big Endian), 22050 Hz, Mono.

visualIamb.mov contains an example of the visual track of an iambic non-sense phrase. Mov format, compression H.264, 25.66 fps, dimensions 320 x 240 pixels.

visualTrochee.mov contains an example of the visual track of a trochaic non-sense phrase. Mov format, compression H.264, 25.66 fps, dimensions 320 x 240 pixels.
